# Host genetics and environment shape the gut microbiome of *Euschistus heros* and *Piezodorus guildinii* and potentially influencing their adaptation

**DOI:** 10.3389/fmicb.2026.1782301

**Published:** 2026-05-05

**Authors:** Matheus Sartori Moro, Thiago Deomar Ludwig, Matheus Scaketti, Ana Flávia Francisconi, Lucas William Mendes, José Baldin Pinheiro, Maria Imaculada Zucchi

**Affiliations:** 1 Departamento de Genética, Escola Superior de Agricultura “Luiz de Queiroz” (ESALQ), Universidade de São Paulo (USP), Piracicaba, Brazil; 2 Centro de Energia Nuclear na Agricultura (CENA), Universidade de São Paulo (USP), Piracicaba, Brazil

**Keywords:** agricultural pests, ecological genomics, insect adaptation, insect gut microbiome, microbial symbiosis

## Abstract

**Background:**

*Euschistus heros* and *Piezodorus guildinii* are major soybean pests across the Americas. Gut-associated bacteria influence insect nutrition, detoxification, and stress tolerance, potentially enhancing adaptation to diverse hosts and management regimes. We investigated how host genetics and environment shape gut microbiome structure and functional potential in these species.

**Results:**

We sequenced the 16S rRNA V4 region from 189 insects collected across Brazil and the United States. Microbiomes were dominated by Proteobacteria and Firmicutes, exhibiting high intra- and interpopulation variability. Diversity and community structure varied significantly among lineages and locations; while environment explained a larger share of overall variance, host genetics provided a more distinct statistical signal for group differentiation. In *E. heros*, genera linked to nutrient acquisition and detoxification (e.g., *Pantoea*, *Wolbachia*) were more prevalent. In *P. guildinii*, *Candidatus Benitsuchiphilus*—associated with diapause and uric-acid metabolism—predominated. Predicted functions included chemoheterotrophy, nitrogen fixation, and degradation of aromatic compounds, with distinct patterns across species and geographic lineages.

**Conclusion:**

Both genetic and environmental factors shape the composition and functional capacity of stink bug gut microbiomes, potentially contributing to host adaptation across different agricultural landscapes. These insights open avenues for microbiome-informed strategies to improve the sustainability and efficacy of soybean pest management.

## 1 Introduction

Hemipteran stink bugs such as *Euschistus heros* and *Piezodorus guildinii* are native to the Neotropics and historically fed on a wide range of native host plants, including weeds and cultivated or wild Fabaceae species (Schaefer and Panizzi, 2000a; Panizzi et al., 2012; Panizzi and Lucini, 2022b). The expansion of soybean cultivation in Brazil from the 1960s onward provided a highly suitable and abundant host, leading to marked population increases and the establishment of these species as major soybean pests (Spehar, 1995; Possebom et al., 2020; Panizzi et al., 2022). Although these insects remain polyphagous, soybean represents their primary host, particularly during reproductive stages when nymphs and adults perform better on immature pods, resulting in population peaks during pod development and filling (Panizzi and Slansky, 1991; Corrêa-Ferreira and De Azevedo, 2002; Oliveira and Panizzi, 2018).

Once established in soybean fields, nymphs and adults feed by inserting their stylets into pods and seeds, injecting digestive enzymes and extracting nutrients (Schaefer and Panizzi, 2000b; Gullan and Cranston, 2014). This feeding behavior causes direct damage to grains, leading to yield losses and reduced seed quality, including lower germination rates, decreased seedling vigor, and altered seed composition. In addition to direct damage, stink bugs may also transmit plant pathogens, resulting in indirect losses to grain quality and seed health (Foster and Daugherty, 1969; Turnipseed and Kogan, 1976; Husseneder et al., 2016).

Chemical control remains the primary management strategy for stink bugs; however, populations of *E. heros* have evolved resistance or reduced susceptibility to multiple insecticides, resulting in variable control efficacy over time (Sosa-Gomez et al., 2001; Sosa-Gómez and da Silva, 2010; Castellanos et al., 2019). Exposure to recurrent environmental and chemical stressors, combined with large population sizes and high reproductive rates, has facilitated the persistence of these species under adverse conditions (Gupta and Nair, 2020). Insects are also known to maintain long-term associations with diverse microorganisms, and accumulating evidence suggests that gut microbiota composition may be associated with host performance under different environmental conditions, although the functional significance of these associations often remains unresolved (Deutsch et al., 2018; Gupta and Nair, 2020).

Stink bugs harbor diverse gut-associated bacterial communities, as demonstrated by both culture-dependent approaches and 16S rRNA surveys in *E. heros* and *P. guildinii*. These studies have reported bacterial taxa belonging mainly to Enterobacteriaceae, Bacillaceae, and Acetobacteraceae, among other groups commonly associated with insect digestive systems (Scopel and Cônsoli, 2018; Franić et al., 2023). Beyond commensal associations, experimental evidence from insect–microbe systems indicates that bacterial interactions may encompass a wide spectrum of ecological outcomes, including pathogenic and insecticidal effects, underscoring the functional heterogeneity of microorganisms associated with phytophagous insects (Husseneder et al., 2016; Nanzer et al., 2021).

Despite these advances, most available studies have focused on single populations, restricted geographic scales, or specific tissues, without explicitly integrating host genetic background and environmental variation in a unified analytical framework (Scopel and Cônsoli, 2018). For major soybean pests such as *E. heros* and *P. guildinii*, large-scale assessments that jointly evaluate gut microbiome composition in relation to insect genotype and environmental context across multiple regions remain scarce. Moreover, functional interpretations derived from 16S rRNA datasets are inherently predictive and taxonomically constrained, underscoring the need for cautious interpretation when linking microbiome composition to adaptive processes (Moro et al., 2021). Consequently, the relative contributions of host genetics and environmental factors in shaping gut microbiome structure in these species are still poorly understood.

This study aims to characterize the diversity and composition of the gut microbiome of the two main soybean stink bug species, *E. heros* and *P guildinii*, to address the following questions: (1) Does insect genotype influence microbiome composition? (2) Is there a pattern in microbiome composition related to the environment? (3) Are microbial taxa associated with functional traits potentially related to host adaptation? Answering these questions may help elucidate the potential influence of genetics and the environment on microbiome modulation and how predicted microbial functions may correlate with insect adaptation to different environments and management conditions. We hypothesize that (1) microbiota composition is influenced by insect genotype, favoring the persistence of certain microorganisms; (2) insects from the same population share more similar microbiota; and (3) microbial taxa show associations with predicted functional traits that may be linked to host adaptation.

## 2 Materials and methods

### 2.1 Insect collection and dissection

Host genetic groups were defined based on previously described population structure for Euschistus heros. Previous population genomic analyses using SNP markers (GBS) identified two major genetic lineages (northern and southern) and a hybrid zone in central Brazil (Zucchi et al., 2019a).

In this study, individuals were assigned to these genetic groups according to their geographic origin and the established distribution of these lineages across Brazil. Specifically, individuals from central regions were classified as hybrid groups, reflecting admixture between northern and southern lineages. This classification follows the population structure framework described by Zucchi et al. (2019a).

Adults of *E. heros* were collected from soybean fields at the reproductive stage in 11 locations in Brazil. Similarly, adults of *P. guildinii* were collected from three locations in Brazil and four locations in the United States. Immediately after collection, insects were euthanized by freezing and stored in Falcon tubes containing 70% ethanol at −20 °C for preservation (Supplementary Table 1).

Dissections were performed by removing the heads, and digestive tracts were collected, including foregut, midgut, and hindgut with attached Malpighian tubules, using sterile forceps. The samples were stored in 1.5 mL microcentrifuge tubes at −20 °C until DNA extraction.

### 2.2 DNA extraction

Bacterial DNA was extracted using a modified CTAB method as follows. Digestive tract samples were placed in 1.5 mL microtubes containing 350 μL of 2% CTAB extraction buffer (20 mM EDTA, 0.1 M Tris–HCl pH 8.0, 1.4 M NaCl, 2% CTAB, plus 0.4% *β*-mercaptoethanol added just before use), 10 μL Proteinase K, and five stainless steel beads (2.8 mm). An additional 350 μL of 2% CTAB was added per tube, and the solution was incubated at 65 °C for 60 min, with inversion every 15 min to aid maceration.

Subsequently, 600 μL of chloroform–isoamyl alcohol (24:1) was added, mixed gently for 1 min, and centrifuged at 10,000 rpm for 15 min. A 600 μL aliquot of the supernatant was transferred to a fresh tube containing 350 μL of cold isopropanol (−20 °C), mixed by inversion, and incubated at −20 °C for 60 min. Samples were then centrifuged at 14,000 rpm for 10 min. The DNA pellet was visible at the bottom of the tube.

The supernatants were removed, and the pellets were washed with 1,000 μL of 70% ethanol and centrifuged at 14,000 rpm for 5 min. After discarding the ethanol, 500 μL of 100% ethanol was added, followed by centrifugation at 14,000 rpm for 10 min. Ethanol was discarded, and tubes were air-dried inverted over sterile filter paper in a biosafety cabinet for at least 3 min. Pellets were resuspended in 50 μL TE buffer (10 mM Tris–HCl pH 7.6, 1 mM EDTA pH 7.6) plus 2 μL RNase (20 mg/mL), incubated at 37 °C for 1 h, and stored at −20 °C (Doyle and Doyle, 1987).

### 2.3 PCR amplification and sequencing

To characterize the microbiome in the bacterial domain, the primer pair 515F and 806R was used to amplify the V4 region of the 16S rRNA gene, generating a 292 bp amplicon. PCR reactions were performed on a high-throughput Fluidigm PCR platform (Biomark™ HD system) at the Roy J. Carver Biotechnology Center, University of Illinois, following Muturi et al. (2017). DNA samples were diluted to 2 ng/μL prior to amplification and processed with the Roche High Fidelity Fast Start Kit and 20 × Access Array Loading Reagent, according to Fluidigm protocols.

Two sets of primers were used in each reaction. The first set included the Fluidigm-specific primers CS1 and CS2 added to the 5’ ends of all ribosomal-specific primers. The second set included the same Fluidigm-specific primers attached to the Illumina i5 primer and barcoded i7 primer. All primers were synthesized by IDT Corp. (Coralville, IA, United States).

The master mix was aliquoted into 48 wells of a PCR plate. To each well, 1 μL DNA sample and 1 μL Fluidigm Illumina linkers with unique barcodes were added. On a separate plate, primer pairs were prepared and aliquoted. The 20 × primer solutions contained 2 μL of each forward and reverse primer (50 μM), 5 μL of 20 × Access Array Loading Reagent, and water to a final volume of 100 μL. The final primer concentration in reactions was 50 nM each.

Samples (4 μL each) were loaded into sample inlets, and 4 μL of primer mix was loaded into primer inlets of a primed Fluidigm 48.48 Access Array IFC. The IFC was placed in the Juno microfluidic system (Fluidigm Corp.) for primer/sample loading, amplification, and harvest. Amplicons were checked on a Fragment Analyzer (Advanced Analytics, Ames, IA, United States) to confirm size and quality.

Amplicons were pooled in equimolar amounts, size-selected on a 2% agarose E-gel (Life Technologies) and extracted with a Qiagen Gel Extraction Kit (Qiagen) using a QIAcube robot. Products were analyzed on an Agilent Bioanalyzer to confirm profiles and determine average size.

A total of 288 samples (286 insects and two water negative controls) were sequenced on two MiSeq flow cells (Illumina) with 301 cycles per read, using a MiSeq 600-cycle kit v3. Reads were 300 nt in length. FASTQ files were demultiplexed using bcl2fastq v2.17.1.14 (Illumina).

Data are available in the NCBI Sequence Read Archive under PRJNA764175 (*E. heros*) and PRJNA764176 (*P. guildinii*).

### 2.4 Sequence data processing

Bacterial (16S rRNA) sequences were processed using the Divisive Amplicon Denoising Algorithm 2 (dada2 package v1.22.0 in R) (Callahan et al., 2016), which provides high sensitivity and resolution (Prodan et al., 2020). Parameters were adapted from the tutorial by Benjamin Callahan^1^ to fit our dataset. Raw reads were quality-filtered and trimmed using filterAndTrim [truncLen = c(250, 180), maxN = 0, maxEE = c(2, 2), truncQ = 2], followed by learning of error rates through an iterative process implemented in learnErrors. Sequences were dereplicated to identify unique reads and their abundances (derepFastq), and forward and reverse reads were merged with mergePairs, retaining only those with sufficient overlap. Chimeric sequences were removed using removeBimeraDenovo with both consensus and sequence-based detection methods. Taxonomic assignment was performed using the RDP Naive Bayesian Classifier trained with the SILVA database (McLaren and Callahan, 2021) for bacterial sequences.

### 2.5 Data analysis

Alpha diversity was assessed to compare the diversity of stink bug microbiomes using the estimate_richness function in the Phyloseq package (McMurdie and Holmes, 2013) on rarefied datasets obtained with rarefy_even_depth. Diversity metrics included Chao1 richness and Shannon diversity indices. Differences among groups were tested using the non-parametric Kruskal–Wallis test, followed by pairwise Wilcoxon rank-sum tests with Bonferroni correction. Beta diversity, used to evaluate differences in community structure among clusters, was quantified by Bray–Curtis dissimilarity and visualized through Principal Coordinate Analysis (PCoA) using the ordinate and plot_ordination functions in Phyloseq. Statistical significance in community composition between clusters was tested using a two-way permutational multivariate analysis of variance (PERMANOVA).

To account for differences in sequencing depth, all samples were rarefied to a minimum threshold of 1,000 reads per sample prior to diversity analyses. This value was chosen because it ensured that all samples retained for analysis were above the threshold while effectively capturing the microbial richness, as demonstrated by the plateau in the rarefaction curves.

To explore microbiome composition, abundance data were transformed to relative abundances (transform with “compositional” method in Phyloseq), after which rare taxa were aggregated using a detection threshold of 5% and a prevalence cutoff via aggregate_rare. Compositional bar plots were generated with the plot_composition function in the Microbiome package (Lahti and Shetty, 2017). The core microbiome was identified using the plot_core function in Microbiome, with a minimum prevalence threshold of 10%.

Putative functional profiles of bacterial communities were predicted using the Functional Annotation of Prokaryotic Taxa (FAPROTAX) database (Louca et al., 2016; Matchado et al., 2024), which maps taxonomic assignments to ecological functions based on curated descriptions of cultured and annotated microorganisms. Functional tables were converted to relative abundances for downstream statistical analysis.

Bacterial community tables, as well as the predicted functional profiles, were analyzed in the Statistical Analysis of Metagenomic Profiles (STAMP) software (Parks et al., 2014) to identify statistically significant differences in taxonomic composition and functional potential. Significance testing was conducted using the two-sided Tukey–Kramer method to calculate *p*-values.

Finally, Spearman’s rank correlation coefficients were computed to evaluate relationships between bacterial abundances and bioclimatic variables, using the corrplot package (Wei and Simko, 2017). Multiple testing correction was applied using the Benjamini–Hochberg false discovery rate procedure (Benjamini and Hochberg, 1995).

## 3 Results

### 3.1 Sequences and samples quality control

**Figure 1:**
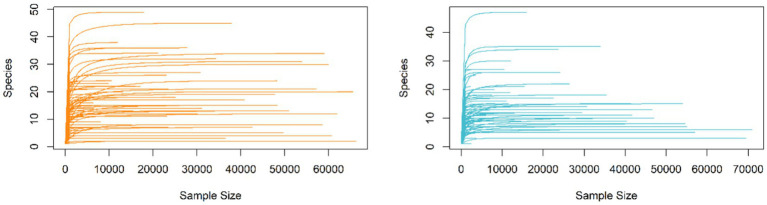
Bacterial rarefaction curve of the gut microbiome of *Euschistus heros* (left) and *Piezodorus guildinii* (right) using Chao1 index.

For the 189 stink bug PCR samples of the bacteria 16S rRNA V4 sequence, Illumina MiSeq sequencing produced 4 million reads (ranging from 3,456 to 53,929 per sample), and the stringency of filtering resulted in high-quality sequence data for subsequent analyses (Weiss et al., 2017). After Amplicon Sequence Variant (ASV) identification, low-quality samples were filtered out for downstream analysis, leaving a total of 96 samples from *E. heros* collected from 10 sites in Brazil and 93 samples from *P. guildinii* collected from three sites in Brazil and four sites in the United States. The depth of sequencing was deemed sufficient, as shown by the plateau in the rarefaction plot reached at 1,000 reads. Although a total of 1,031 ASVs were identified, the early stabilization of the rarefaction curves indicates that the low number of observed ASVs is a biological characteristic of the stink bug gut microbiome—often dominated by specific symbionts—rather than an artifact of low sequencing depth (Figure 1).

### 3.2 Microbiome structure and diversity

**Figure 2:**
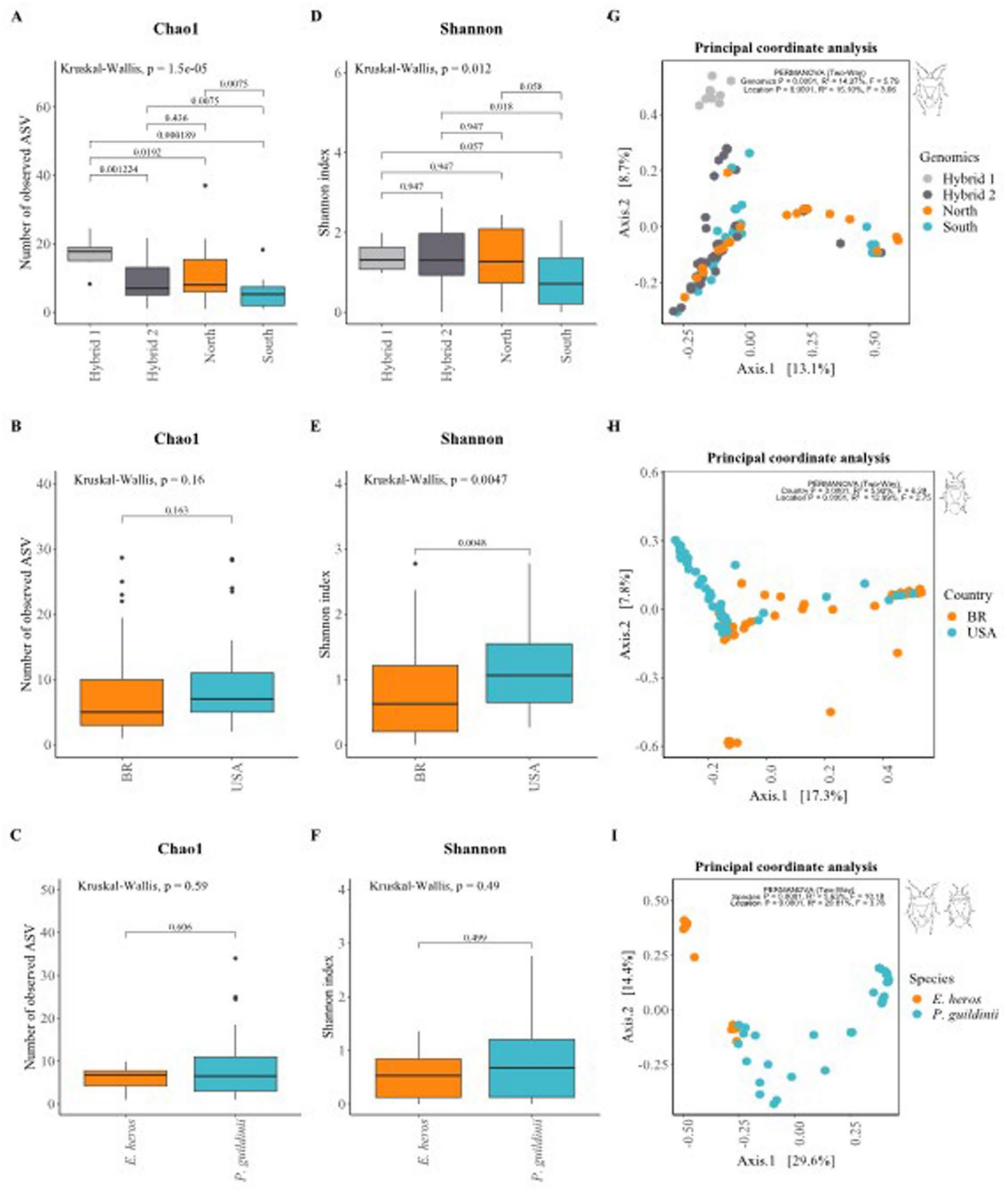
Diversity and community structure of the gut microbiome of *E. heros* and *P. guildinii*. **(A–C)** *α*-Diversity based on the Chao1 index for *E. heros* **(A)**, *P. guildinii* **(B)**, and both species combined **(C)**. **(D–F)** α-Diversity based on the Shannon index for *E. heros* **(D)**, *P. guildinii* **(E)**, and both species combined **(F)**. Boxplots are shown at the ASV level, grouped by lineage or country. Kruskal–Wallis test *p*-values are indicated in the upper left of each panel, and pairwise comparisons (Wilcoxon–Mann–Whitney test with Benjamini–Hochberg correction) are shown between groups. **(G–I)** *β*-Diversity based on Bray–Curtis dissimilarity and visualized with Principal Coordinates Analysis (PCoA) for *E. heros* **(G)**, *P. guildinii* **(H)**, and both species combined **(I)**. PERMANOVA results are reported in the upper right corner of each plot, including *R*^2^ values and significance levels (*p* = 0.0001).

The *α*-diversity analysis measures within-sample diversity. For *E. heros*, intestinal microbiome diversity varied among lineages for both Chao1 (Figure 2A; Kruskal–Wallis, *p* < 0.0001) and Shannon indices (Figure 2B; Kruskal–Wallis, *p* < 0.05). The Chao1 index, reflecting species richness, was highest in insects from the first hybrid group, followed by the second hybrid group and the northern lineage, with the southern lineage showing the lowest richness. For the Shannon index, which accounts for both richness and evenness, insects from the second hybrid group displayed greater evenness compared with those from the southern lineage (Figure 2B; Wilcoxon, *p* = 0.018).

For *P. guildinii*, species richness was similar among countries (Figure 2B; Kruskal–Wallis, *p* > 0.05), but evenness was higher in insects from the United States (Figure 2E; Kruskal–Wallis, *p* < 0.05). Similarly, when comparing both species, richness did not differ significantly between countries (Figure 2C; Kruskal–Wallis, *p* > 0.05), whereas evenness was greater in samples from the United States (Figure 2F; Kruskal–Wallis, *p* < 0.05).

The *β*-diversity analysis compares the variation between samples according to lineage. Although the Principal Coordinates Analysis (PCoA) did not reveal clear clustering among lineages, significant dissimilarities were observed (PERMANOVA, *p* = 0.0001). For *E. heros*, both host genomics (*R*^2^ = 14.27%, *F* = 5.79, *p* = 0.0001) and location (*R*^2^ = 15.10%, *F* = 3.06, *p* = 0.0001) significantly influenced microbiome structuring (Figure 2G). While genomics yielded a higher *F*-value, indicating stronger statistical separation, location explained slightly more variance. For *P. guildinii*, a slight but significant structuring was observed between countries (PERMANOVA, *p* = 0.0001). In this case, host genomics (*R*^2^ = 5.92%, *F* = 6.28, *p* = 0.0001) produced a stronger statistical signal, whereas environment accounted for a larger share of variance (*R*^2^ = 12.99%, *F* = 2.75, *p* = 0.0001) (Figure 2H). When comparing *E. heros* and *P. guildinii*, a modest but significant structuring of the gut microbiome was observed (PERMANOVA, *p* = 0.0001). Species explained a small proportion of the variance (*R*^2^ = 5.63%, *F* = 10.18, *p* = 0.0001), whereas location accounted for a much larger share (*R*^2^ = 20.81%, *F* = 3.76, *p* = 0.0001). Thus, although the F-value was higher for species, indicating a stronger statistical separation, environment was more important in structuring the microbiome (Figure 2I).

### 3.3 Microbiome composition of *E. heros* and *P. guildinii*

**Figure 3:**
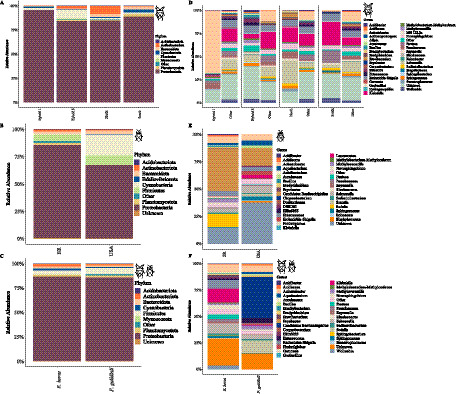
Relative abundance of gut bacterial communities of *E. heros* and *P. guildinii*. **(A)** Composition of *E. heros* across hybrid groups and geographic lineages at the phylum level. **(B)** Composition of *P. guildinii* from Brazil (BR) and the United States (USA) at the phylum level. **(C)** Comparative composition of *E. heros* and *P. guildinii* at the phylum level. **(D)** Relative abundance of bacterial genera in *E. heros* across hybrid groups and lineages. **(E)** Relative abundance of bacterial genera in *P. guildinii* from Brazil and the United States. **(F)** Comparative composition of *E. heros* and *P. guildinii* at the genus level.

The gut microbiomes of both stink bug species were predominantly composed of Proteobacteria, which accounted for an average abundance of 86.62% in *E. heros* and 90.84% in *P. guildinii*. Other shared phyla included Firmicutes, Actinobacteriota, and Bacteroidota, collectively representing over 97% of the bacterial community in both hosts (Figures 3A–C; Supplementary Figure S1).

Despite these broad similarities at the phylum level, significant differences emerged in the distribution of key bacterial genera (Figure 3F). *P. guildinii* was characterized by the high prevalence of the specialized symbiont *Candidatus Benitsuchiphilus* (47.54%) and *Aquabacterium*, which were significantly more abundant than in *E. heros*. In contrast, *E. heros* displayed a more diverse array of prevalent genera, with *Escherichia/Shigella*, *Klebsiella* (13.03%), *Salmonella* (9.01%), *Pantoea*, and *Acinetobacter* (6.95%) being significantly more predominant than in *P. guildinii* (Figure 3F).

Furthermore, several taxa were species-specific; for instance, *Aureimonas*, *Brachybacterium*, *Brevibacterium*, *Corynebacterium*, *Ellin6055*, *Rhodococcus*, *Sphingobacterium*, *Stenotrophomonas*, and *Wolbachia* were unique to *E. heros*, whereas *Sodalis* and *Fimbriiglobus* were unique to *P. guildinii* (Figure 3E). Within *E. heros* lineages, specific associations were also observed: the northern lineage showed a higher abundance of *Brevibacterium* and *Pseudomonas*, while the southern lineage was enriched with *Klebsiella* and *Pantoea* (Figure 3D). These compositional differences highlight species-specific recruitment and maintenance of gut microbial communities despite sharing similar soybean-producing environments.

### 3.4 Core microbiome composition of *Euschistus heros* across lineages and hybrids

**Figure 4:**
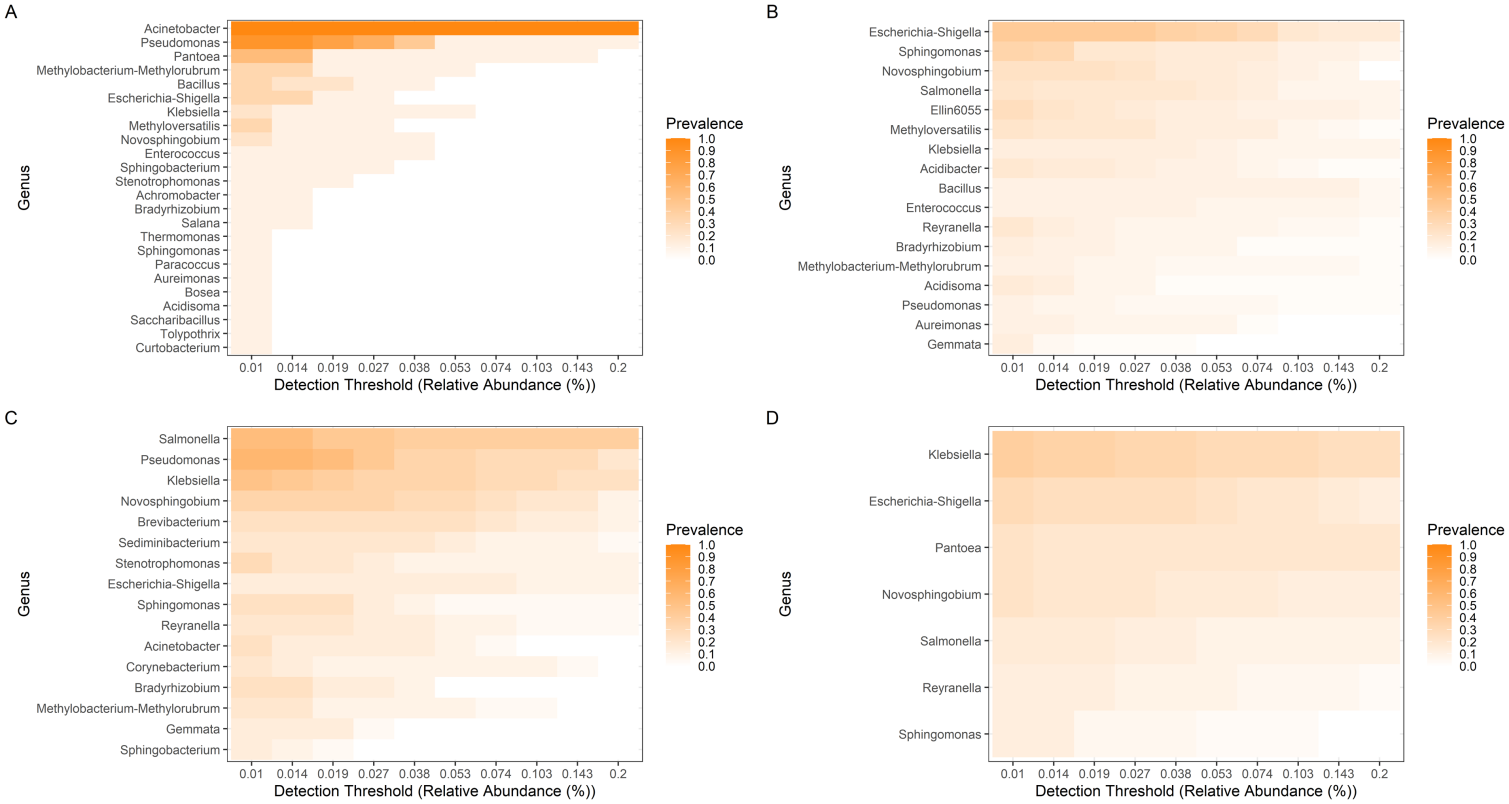
Core bacterial genera in the gut microbiome of *E. heros*. The prevalence of bacterial genera is shown for **(A)** Hybrid 1, **(B)** Hybrid 2, **(C)** Northern lineage, and **(D)** Southern lineage. Color intensity represents the proportion of individuals in which each genus was detected, across increasing thresholds of relative abundance.

Although *E. heros* individuals were collected from the same locations, their gut microbiota varied within populations (Figure 4A). In Hybrid 1, *Acinetobacter* and *Pseudomonas* were highly prevalent (Figure 4A), whereas *Escherichia/Shigella* showed lower prevalence in Hybrid 2 (Figure 4B). In the northern lineage, *Salmonella*, *Pseudomonas*, and *Klebsiella* were frequent (Figure 4C), while *Klebsiella* dominated in the southern lineage (Figure 4D). Across all groups, *Klebsiella*, *Escherichia/Shigella*, *Novosphingobium*, and *Sphingomonas* were consistently detected (Figure 4).

**Figure 5:**
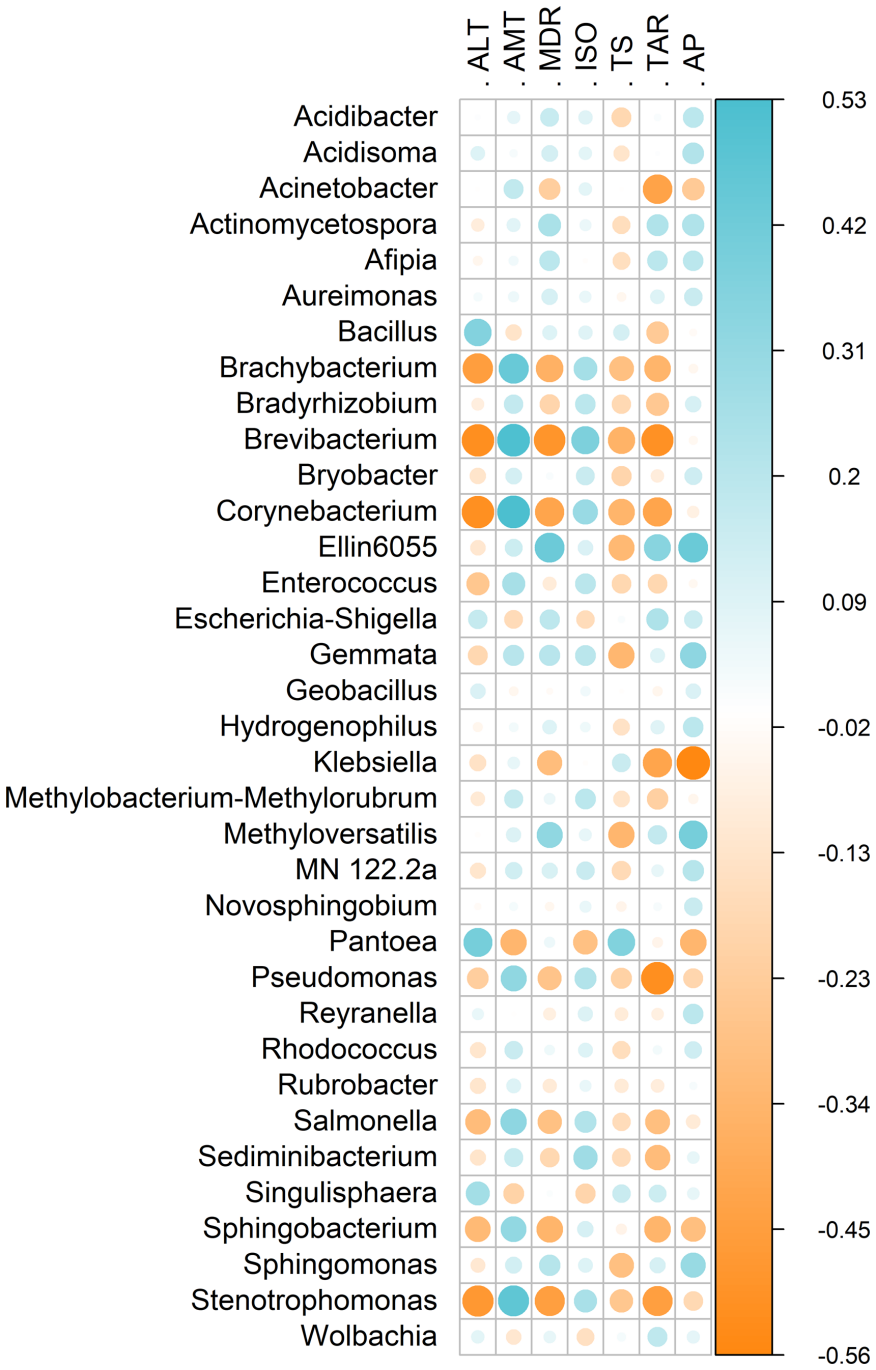
Heatmap showing Spearman’s rank correlation coefficients between bacterial genera of *E. heros* and environmental variables. Blue and orange colors indicate significant positive and negative correlations, respectively (*p* < 0.05). Only significant correlations are displayed. Environmental variables: altitude (ALT), annual mean temperature (AMT), mean diurnal range (MDR), isothermality (ISO), temperature seasonality (TS), temperature annual range (TAR), and annual precipitation (AP).

Environmental factors were significantly correlated with the gut microbiota of *E. heros* (Figure 5). Spearman’s correlations indicated strong associations for several genera, including *Brachybacterium*, *Brevibacterium*, *Corynebacterium*, *Ellin6055*, *Klebsiella*, *Pantoea*, *Pseudomonas*, *Salmonella*, *Sphingobacterium*, and *Stenotrophomonas*. Genera predominant in northern lineages and hybrids (*Acinetobacter*, *Ellin6055*, *Brevibacterium*, *Pseudomonas*, *Salmonella*) showed negative correlations with temperature seasonality (TS), whereas genera prevalent in the southern lineage (*Klebsiella* and *Pantoea*) were positively correlated with this variable.

### 3.5 Core microbiome composition of *Piezodorus guildinii* across geographic populations

**Figure 6:**
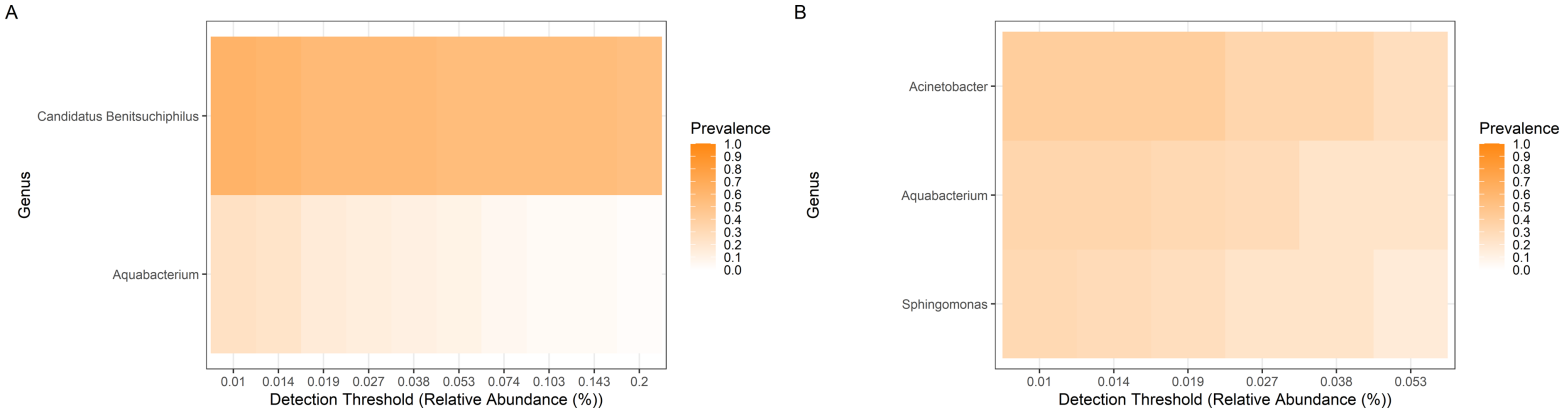
Core bacterial genera in the gut microbiome of *P. guildinii*. The prevalence of bacterial genera is shown for **(A)** Brazilian populations and **(B)** United States populations. Color intensity represents the proportion of individuals in which each genus was detected, across increasing thresholds of relative abundance.

Although *P. guildinii* individuals were collected from the same locations, their gut microbiota varied within populations (Figure 6). In Brazilian populations, *Candidatus Benitsuchiphilus* and *Aquabacterium* were prevalent (Figure 6A), whereas in U. S. populations, *Acinetobacter*, *Aquabacterium*, and *Sphingomonas* were detected (Figure 6B). *Aquabacterium* was observed in all individuals, although at low prevalence.

**Figure 7:**
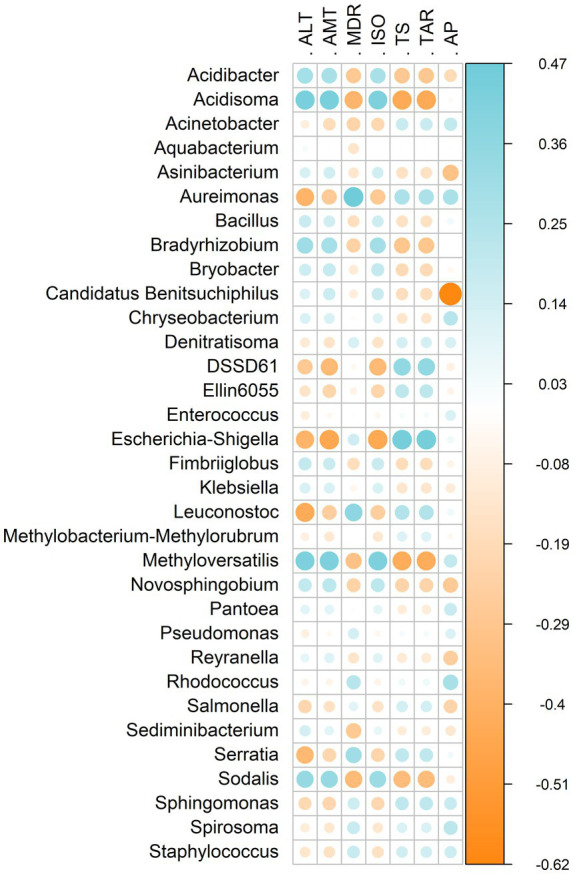
Heatmap showing Spearman’s rank correlation coefficients between bacterial genera of *P. guildinii* and environmental variables. Blue and orange colors indicate significant positive and negative correlations, respectively (*p* < 0.05). Only significant correlations are displayed. Environmental variables: altitude (ALT), annual mean temperature (AMT), mean diurnal range (MDR), isothermality (ISO), temperature seasonality (TS), temperature annual range (TAR), and annual precipitation (AP).

Environmental factors were significantly correlated with the gut microbiota of *P. guildinii* (Figure 7). Strong correlations were observed for *Acidibacter*, *Acidisoma*, *Aureimonas*, *Escherichia/Shigella*, *Methyloversatilis*, and *Sodalis*. Genera prevalent in Brazilian populations (*Acidibacter*, *Candidatus Benitsuchiphilus*, *Novosphingobium*, *Sodalis*) showed positive correlations with altitude (ALT), annual mean temperature (AMT), and isothermality (ISO), but negative correlations with mean diurnal range (MDR), temperature seasonality (TS), temperature annual range (TAR), and annual precipitation (AP). In contrast, genera prevalent in North American populations (*Pseudomonas*, *Sphingomonas*) displayed the opposite pattern.

### 3.6 Comparative core microbiome of *Euschistus heros* and *Piezodorus guildinii*

**Figure 8:**
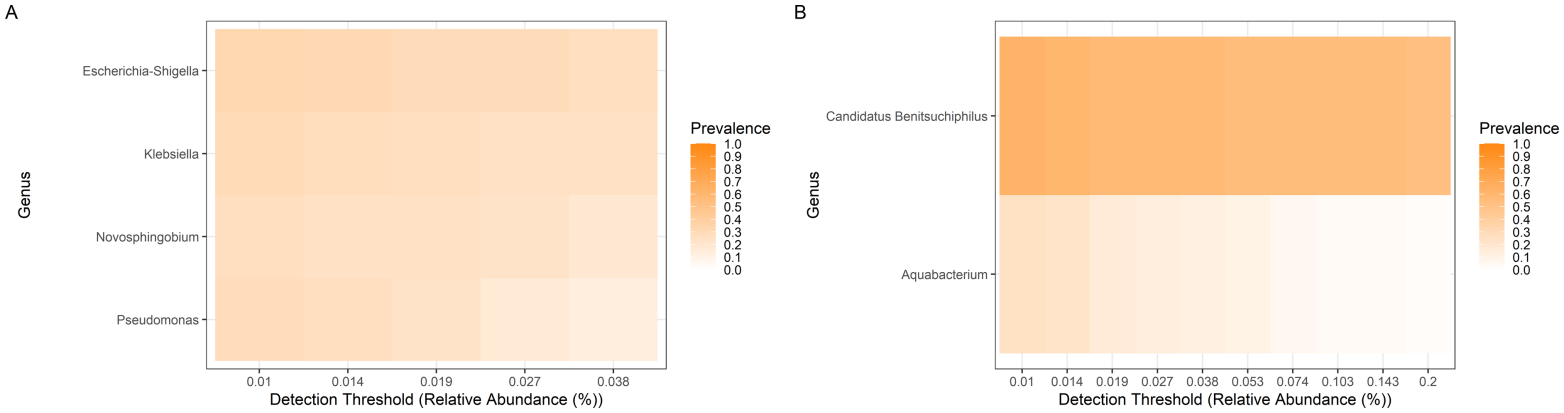
Core bacterial genera in the gut microbiome of Brazilian populations. The prevalence of bacterial genera is shown for **(A)** *E. heros* and **(B)** *P. guildinii*. Color intensity represents the proportion of individuals in which each genus was detected, across increasing thresholds of relative abundance.

*Euschistus heros* and *Piezodorus guildinii* exhibited distinct bacterial communities within the same population, even though individuals were collected from the same locality. Despite high variation in microbiome composition, some genera were consistently recruited by all individuals of each species. In Brazilian *E. heros*, the core included *Escherichia/Shigella*, *Klebsiella*, *Novosphingobium*, and *Pseudomonas* (Figure 8A), while in Brazilian *P. guildinii* the genera *Candidatus Benitsuchiphilus* and *Aquabacterium* were predominant (Figure 8B). Thus, each species harbors distinct core genera.

**Figure 9:**
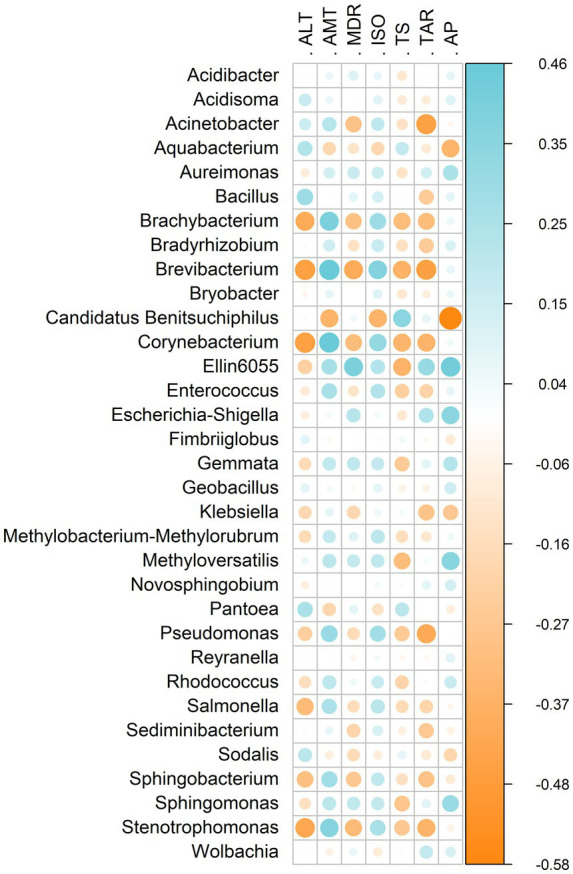
Heatmap showing Spearman’s rank correlation coefficients between bacterial genera of *E. heros* and *P. guildinii* and environmental variables. Blue and orange colors indicate significant positive and negative correlations, respectively (*p* < 0.05). Only significant correlations are displayed. Environmental variables: altitude (ALT), annual mean temperature (AMT), mean diurnal range (MDR), isothermality (ISO), temperature seasonality (TS), temperature annual range (TAR), and annual precipitation (AP).

Environmental factors were significantly correlated with the gut microbiota of *E. heros* and *P. guildinii* (Figure 9). Strong associations were detected for *Brachybacterium*, *Brevibacterium*, *Candidatus Benitsuchiphilus*, *Corynebacterium*, *Ellin6055*, and *Stenotrophomonas*. In *E. heros*, prevalent genera (*Aureimonas*, *Brevibacterium*, *Corynebacterium*, *Ellin6055*, *Escherichia/Shigella*, *Klebsiella*, *Pantoea*, *Pseudomonas*, *Reyranella*, *Sphingomonas*, *Wolbachia*) were positively correlated with annual mean temperature (AMT), isothermality (ISO), and annual precipitation (AP), and negatively correlated with altitude (ALT) and temperature seasonality (TS). In contrast, *Aquabacterium*, *Candidatus Benitsuchiphilus*, and *Sodalis*, predominant in *P. guildinii*, showed the opposite pattern.

### 3.7 Microbiome functions of *Euschistus heros*

**Figure 10:**
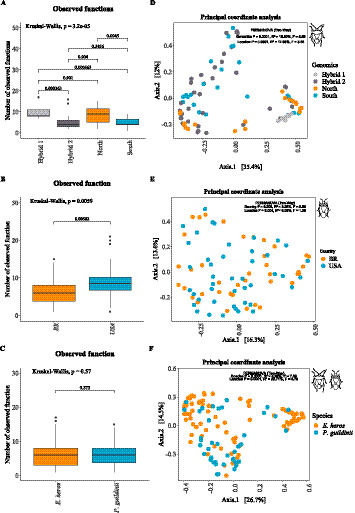
The predicted functional profile of the gut microbiome of *E. heros* and *P. guildinii* revealed significant differences according to host lineage, country, and species. For *E. heros*, the number of observed functions varied significantly among lineages, with higher diversity in Hybrid 1 and northern insects compared to Hybrid 2 and southern insects [Kruskal–Wallis, *p* < 0.0001; **(A)**]. In *P. guildinii*, functional diversity also differed between countries, being higher in individuals from the United States than in those from Brazil [Kruskal–Wallis, *p* = 0.0059; **(B)**]. However, when comparing both species, the number of observed functions was similar [Kruskal–Wallis, *p* = 0.57; **(C)**]. β-diversity analyses indicated that although no clear clustering was observed, significant dissimilarities existed between lineages, countries, and species, with variation explained both by host genetics and geography [PERMANOVA, *p* = 0.0001; **(D–F)**].

After characterizing the gut microbiome diversity and composition of *E. heros*, we next assessed the potential functions performed by the bacteria and their role in the adaptation of these stink bugs to soybean fields and new hosts. The *α*-diversity analysis revealed significant differences in the number of observed functions among lineages (Figure 10A; Kruskal–Wallis, *p* < 0.0001). Insects from the first hybrid group exhibited the highest number of functions, followed by those from the northern lineage, with the second hybrid group and the southern lineage showing lower functional diversity. Although the PCoA did not display a clear clustering of lineages, functional profiles differed significantly among groups (PERMANOVA, *R*^2^ = 15.89%, *F* = 6.55, *p* = 0.0001), with the two main axes explaining 47.4% of the variation in bacterial functions (Figure 10D). These results indicate that lineage effects produced stronger statistical separation, whereas environmental factors explained a larger proportion of the variance (*R*^2^ = 17.85%, *F* = 3.68, *p* = 0.0001) in shaping the functional potential of the gut microbiome.

### 3.8 Microbiome functions of *Piezodorus guildinii*

After jointly assessing the gut microbiome diversity and composition of *E. heros* and *P. guildinii*, we further investigated the potential functions performed by their associated bacteria and how they differ between species. The α-diversity analysis revealed no significant differences in the number of observed functions between species (Figure 10B; *p* = 0.57). However, the *β*-diversity analysis based on PCoA showed that, despite the absence of clear clustering, functional profiles were significantly structured by environmental factors. PERMANOVA indicated significant effects of country (*R*^2^ = 2.92%, *F* = 2.56, *p* = 0.003) and sampling location (*R*^2^ = 9.09%, *F* = 1.59, *p* = 0.004) (Figure 10E), suggesting that environmental variation plays a role in shaping the functional potential of the gut microbiome.

### 3.9 Comparative microbiome functions of *Euschistus heros* and *Piezodorus guildinii*

When comparing *E. heros* and *P. guildinii* together, the α-diversity analysis revealed no significant differences in the number of observed functions between species (Figure 10C; *p* = 0.57). In contrast, the β-diversity analysis based on PCoA revealed significant differences in functional profiles according to both species and location. Species identity explained 4.60% of the variation (*R*^2^ = 4.60%, *F* = 7.59, *p* = 0.0001), whereas location accounted for a substantially larger proportion, explaining 22.71% of the variation (*R*^2^ = 22.71%, *F* = 3.75, *p* = 0.0001; Figure 10F). These results indicate that, although functional richness is similar between species, the composition of predicted bacterial functions differs significantly, with environmental factors exerting a stronger influence than host genotype.

### 3.10 Predicted functional potential of *Euschistus heros*

**Figure 11:**
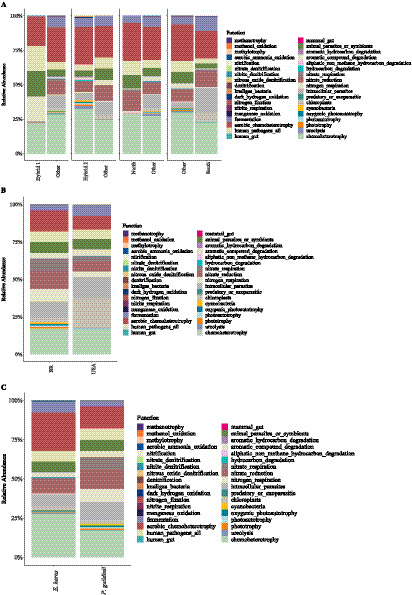
Predicted functional traits of the gut bacterial microbiome of *E. heros* and *P. guildinii*. **(A)** Relative abundance of predicted functional traits across *E. heros* hybrid groups and geographic lineages. **(B)** Relative abundance of predicted functional traits in *P. guildinii* populations from Brazil (BR) and the United States (USA). **(C)** Comparative functional traits of *E. heros* and *P. guildinii*. Functions include processes such as chemoheterotrophy, fermentation, nitrogen fixation, denitrification, ureolysis, degradation of aromatic and aliphatic compounds, and associations with mammal and human gut, parasites, or symbionts. Bars represent the proportion of functional categories predicted for each group, showing differences in the functional potential of gut microbial communities.

The putative functions predicted for the gut bacteria of *E. heros* were potentially related to the use of chemical compounds as energy sources, oxidation and denitrification processes, nitrogen fixation, fermentation, aromatic compound degradation, respiration, phototrophy, and ureolysis. In addition, some bacterial groups commonly found in the intestines of mammals and humans were also identified, predicted to potentially act as either pathogens or symbionts. Chemoheterotrophy was the most frequent inferred process, being consistently present across all lineages (Figure 11A). Inferred functional differences were observed among lineages: the first hybrid group showed potential enrichment in aromatic compound degradation, animal-associated parasites or symbionts, and human pathogens; the second hybrid group exhibited higher frequencies of predicted chemotrophic functions; while the southern lineage was putatively enriched in intracellular parasites. The northern lineage, however, did not differ from the other groups. Notably, inferred functions associated with aromatic hydrocarbon degradation, aliphatic non-methane hydrocarbon degradation, and hydrocarbon degradation were unique to hybrid insects (Figure 11A).

### 3.11 Predicted functional potential of *Piezodorus guildinii*

The putative functions of the gut microbiome in *P. guildinii* were predicted to be broadly similar between countries. The dominant inferred processes included chemoheterotrophy, oxidation and denitrification, nitrogen fixation, fermentation, respiration, phototrophy, and ureolysis (Figure 11B). Chemoheterotrophy was again the most frequent putative function across all samples. However, potential functions related to aromatic hydrocarbon degradation, aliphatic non-methane hydrocarbon degradation, and hydrocarbon degradation were unique to insects collected in the United States, and predicted to be absent in those from Brazil (Figure 11B).

### 3.12 Comparative predicted functional potential between *Euschistus heros* and *Piezodorus guildinii*

When comparing *E. heros* and *P. guildinii*, the predicted functional traits were largely shared, including chemoheterotrophy, oxidation and denitrification, nitrogen fixation, fermentation, respiration, phototrophy, and ureolysis. Chemoheterotrophy remained the most frequent inferred process in both species (Figure 11C). No significant differences were detected in the putative functions shared by the two stink bug species. Nonetheless, predicted functional richness was higher in northern *E. heros* and hybrid lineages compared with southern populations, while *P. guildinii* from the United States displayed higher inferred diversity than those from Brazil. Overall, species identity exerted a stronger influence than environment in shaping predicted functional potential, with *E. heros* harboring greater putative diversity than *P. guildinii*.

## 4 Discussion

This study evaluated the gut microbiome of two major soybean stink bug pests, *E. heros* and *P. guildinii*, describing the structure and composition of bacteria, as well as inferring possible bacterial functions associated with different lineages. Our results support a hierarchical framework in which gut microbiome assembly in stink bugs is shaped by (i) environmental exposure (plants, soil, climate), (ii) host behavior (diet breadth and diapause), and (iii) host filtering mechanisms (gut physiology and immune regulation), all modulated by host genetic background.

While our results demonstrate that both host genetics and location significantly influence microbiome structure, it is important to acknowledge that this study did not explicitly account for seasonal variations or *in situ* environmental factors such as precise temperature and humidity during sampling. The insect gut microbiome is known to be dynamic, and these abiotic variables can potentially introduce biases into microbial diversity estimations. However, to minimize such effects, all samples were collected during the reproductive stage of the insects in soybean fields. Furthermore, we observed significant correlations between several bacterial genera and long-term bioclimatic variables, suggesting that regional climatic patterns, rather than just immediate conditions, play a substantial role in shaping these microbial communities. Future longitudinal studies incorporating real-time environmental monitoring will be valuable to further refine the impact of seasonality on the stink bug microbiome.

### 4.1 Microbiome diversity and community structure

In both species, gut microbiome diversity and structure varied among lineages and locations. PERMANOVA results indicated that environment (location) explained a larger proportion of the total variation in the microbiome (higher R^2^), whereas genetics exhibited higher pseudo-*F* values, indicating stronger separation among genetic groups relative to within-group variability. Thus, both factors play complementary roles: the environment contributes more to the overall variance explained, while host genetics enhances microbiome differentiation among lineages.

The assembly of the gut microbiome can be understood as a two-stage process involving microbial acquisition and colonization. After hatching, stink bug nymphs remain aggregated near egg masses, where they acquire maternally deposited and environmentally derived microorganisms. From the fourth instar onward, nymphs disperse and expand their microbial exposure through interactions with plants, soil, and surrounding substrates (Panizzi and Slansky, 1985; Malaguido and Panizzi, 1999; Shan et al., 2024). The environment likely represents the primary source of microbial acquisition across generations (Engel and Moran, 2013), and the gut microbiome tends to reflect the local environmental microbial pool (Rani et al., 2009), a pattern consistently observed across diverse insect systems (Zouache et al., 2011).

Diet is one of the strongest ecological drivers of gut microbial diversity. Stink bugs feed on a wide range of plants, from weeds to cultivated species (Bernays and Chapman, 1994; Schaefer and Panizzi, 2000b), using plant chemical cues for recognition (Sharma et al., 2021). Plant secondary metabolites such as flavonoids, carotenoids, alkaloids, and other bioactive compounds, can alter gut microbial composition (Martínez-Romero et al., 2021). Feeding allows insects to ingest plant-associated microbes, and the gut environment provides favorable conditions for colonization, including nutrient availability and protection from external stress (Douglas, 2015). Consistent with previous studies, food sources strongly influence microbiome diversity; however, our results further indicate that this effect is modulated by host lineage and regional climatic conditions (Broderick et al., 2004; Priya et al., 2012).

Seasonal survival strategies also shape microbial acquisition. After soybean harvest, stink bugs overwinter in the soil or crop residues for up to 7 months without feeding (Panizzi and Vivan, 1997), which may facilitate contact with soil-associated bacteria. Similar acquisition of soil symbionts was observed in R. clavatus, where Burkholderia was acquired environmentally in the absence of parental transmission (Kikuchi et al., 2007).

Once established, the microbiota becomes subject to host-driven selective pressures. Gut epithelial cells secrete a peritrophic matrix that provides protection against microbial invasion (Engel and Moran, 2013; Gullan and Cranston, 2014; Gupta and Nair, 2020), while the immune system and gut physiology further regulate colonization (Mallott and Amato, 2021). Factors such as oxygen availability, pH, and gut morphology (e.g., specialized caeca in Pentatomids) can influence microbial persistence and are often linked to host genetics (Prado et al., 2006). Some microbes may also transition to intracellular associations and be transmitted vertically, as females deposit symbionts on egg surfaces that are later ingested by nymphs (Panizzi, 1997; Prado and Zucchi, 2012; Engel and Moran, 2013). These mechanisms help maintain stable symbiotic relationships across generations.

Alpha diversity patterns also reflected ecological and genetic differences. In *E. heros*, northern and hybrid lineages showed higher bacterial diversity than southern lineages, likely reflecting both genotype and dietary breadth. Northern populations are more often associated with native plants and beans, whereas southern populations are strongly tied to soybean (2019). Nutrient-rich or homogeneous diets may reduce microbial richness (Erkosar et al., 2018; Kešnerová et al., 2020), while photoperiod-driven reproductive diapause in southern populations (Mourão and Panizzi, 2000), may further reduce feeding activity and, consequently, microbial acquisition.

In *P. guildinii*, higher bacterial diversity was observed in U. S. populations compared to Brazil. This difference may relate to agricultural practices: although pesticide use is comparable in total volume, concentrations applied are higher in Brazil (5–10 kg/ha) than in the U. S. (2.5–5 kg/ha), potentially exerting stronger negative effects on soil microbiota and reducing environmental microbial pools (Meena et al., 2020).

Finally, differences in host behavior may also explain diversity patterns. *E. heros* is reported on at least 15 weed species across 9 families, whereas *P. guildinii* is associated with only 7 weed species across 6 families (Panizzi et al. 2022a). Even when both occur on the same host plants, *E. heros* is found at much higher frequencies (Engel et al., 2019a; Engel et al., 2019b). Thus, despite being polyphagous, *E. heros* exploits a broader and more abundant set of plant hosts, which likely contributes to its richer and more differentiated gut microbiome.

### 4.2 Taxonomic composition and functional roles

Contrary to our second hypothesis, we observed very high variability in bacterial microbiome composition even among individuals of the same population, particularly in insects from Uberlândia/MG. Despite this variation, Proteobacteria and Firmicutes dominated the gut microbiome of both *E. heros* and *P. guildinii*, consistent with previous reports across Hemiptera and other insects (Colman et al., 2012; Jones et al., 2013; Yun et al., 2014; Kim et al., 2017). Several genera within these phyla (e.g., *Acinetobacter*, *Pantoea*, *Pseudomonas*, *Serratia*, *Staphylococcus*) are common plant endophytes and were also recovered here, supporting the view that host plants are the main source of gut microbiota acquisition (Elvira-Recuenco and Van Vuurde, 2000; Kuklinsky-Sobral et al., 2005; Costa et al., 2012; Egamberdieva et al., 2016; De Almeida Lopes et al., 2018; Zhao et al., 2018; Mayhood and Mirza, 2021).

These bacterial groups are known to provide key ecological and adaptive functions, including nutrient supplementation, digestion, immunity, reproduction, and tolerance to environmental stressors (Russell et al., 2014; Douglas, 2015; Hammer and Bowers, 2015; Arbuthnott et al., 2016; Wielkopolan and Obrępalska-Stęplowska, 2016; Engl and Kaltenpoth, 2018; Jing et al., 2020). Symbionts such as *Acinetobacter*, *Staphylococcus*, and *Wolbachia* have recognized roles in nutrient assimilation, reproduction, and adaptation (Shigenobu et al., 2000; Barr et al., 2010; Graham and Wilson, 2012; Chu et al., 2013; Chung et al., 2013; Mason et al., 2016; Xue et al., 2021). Other taxa, including *Pantoea* and *Enterococcus*, can enhance tolerance to soybean chemical defenses and have been reported as vertically transmitted in Pentatomids (Hirose et al., 2006; Medina et al., 2018).

A major adaptive role of the microbiome involves detoxification. Several genera found here (*Acinetobacter, Pantoea, Pseudomonas, Serratia, Stenotrophomonas*) participate in the degradation of plant allelochemicals and insecticides. For instance, *Pseudomonas* strains associated with *P. guildinii* can metabolize aromatic compounds and confer resistance to organophosphate insecticides (van den Bosch and Welte, 2017). Likewise, *Bacillus* species are capable of detoxifying acephate, a key insecticide used against stink bug (Somavilla et al., 2020). Interestingly, *Burkholderia*, a well-documented symbiont mediating insecticide resistance in *Riptortus pedestris* (Jang and Kikuchi, 2020), was not detected here, consistent with the environmental dependence of its transmission (Scopel and Cônsoli, 2018).

The dual role of symbionts must also be considered. While many contribute to host adaptation, others act as pathogens or vectors of plant diseases. For example, stink bugs can transmit *Pseudomonas flectens* or *Serratia marcescens* to crops, turning commensal bacteria into phytopathogens after repeated inoculations (Husseneder et al., 2016). Similarly, entomopathogenic *Bacillus thuringiensis* can disrupt gut cells, leading to host mortality (de Bortoli and Jurat-Fuentes, 2019).

Overall, our findings suggest that beyond basic metabolic roles (oxidation, denitrification, nitrogen fixation), the gut microbiota of *E. heros* and *P. guildinii* is likely contributing to nutrient acquisition, detoxification of phytochemicals and pesticides, and potential pathogen transmission. These functions highlight the dual ecological role of the microbiome in enhancing host adaptation while also representing risks for agriculture.

In the northern lineage of *E. heros*, the genera *Brevibacterium*, *Pseudomonas*, and *Salmonella* were most abundant. The higher prevalence of *Pseudomonas* may be linked to the stronger association of this lineage with native plants (Zucchi et al., 2019b), as members of this genus are involved in phytochemical detoxification (Van den Bosch and Welte, 2017; Itoh et al., 2018). This pattern suggests that microbiome composition may reflect host plant use and associated chemical environments. Although Brevibacterium has been reported as pathogenic to pentatomids (Zucchi et al., 2012), the reason for its higher frequency in this lineage remains unclear.

In contrast, the southern lineage of *E. heros* was dominated by *Klebsiella* and *Pantoea*. The abundance of *Klebsiella* may reflect agricultural practices in southern Brazil, where soybean is rotated with sugarcane to enhance soil nitrogen and manage pests (Pavão et al., 2015). *Klebsiella* var*iicola*, an endophyte of sugarcane roots involved in nitrogen fixation, could be acquired from the soil, while *K. pneumoniae* has previously been isolated from *Nezara viridula* and may have a symbiotic role in stink bugs (Medrano and Bell, 2017). *Pantoea*, in turn, has strong bioremediation potential, including degradation of the herbicide mesotrione widely applied in sugarcane fields (Pileggi et al., 2012). The greater abundance of both genera in the southern lineage may also be influenced by local climatic factors, as they showed a positive correlation with temperature seasonality. Together, these patterns indicate that agricultural systems may indirectly shape gut microbiome composition through environmental filtering and exposure to agrochemicals.

In Brazilian populations of *P. guildinii*, the most abundant genera were *Acidibacter*, *Candidatus Benitsuchiphilus*, *Novosphingobium*, and *Sodalis*. *Candidatus Benitsuchiphilus* is a maternally transmitted symbiont (Hosokawa et al., 2010; Hosokawa et al., 2012) with a long-term evolutionary association, providing essential vitamins, cofactors, and enzymes for uric acid metabolism such as urate oxidase and glutamine synthetase (Mondal et al., 2020). Its higher abundance may reflect a stronger dependence of Brazilian populations on these symbiotic contributions. *Novosphingobium*, in turn, contributes to cellulose and xylan degradation, potentially aiding digestion (Jang and Kikuchi, 2020), and may also be more prevalent due to its natural occurrence in soybean nodules and rhizosphere (Mayhood and Mirza, 2021). These results indicate that both vertical transmission and environmental acquisition jointly contribute to microbiome assembly in this species.

In contrast, North American populations were dominated by *Pseudomonas* and *Sphingomonas*. The prevalence of *Pseudomonas* likely reflects its capacity to detoxify plant secondary metabolites (van den Bosch and Welte, 2017; Itoh et al., 2018) and degrade organophosphate insecticides (Husseneder et al., 2016).

Other genera showed no clear functional association beyond differences in environmental availability. Notably, correlations with bioclimatic variables revealed contrasting patterns between regions, with taxa enriched in Brazilian populations positively associated with certain variables, whereas those dominant in U. S. populations showed negative correlations, and vice versa. These opposing trends further support the role of climatic filtering in structuring gut microbiomes at large geographic scales.

When comparing the two species, *E. heros* harbors a higher abundance of feeding-related bacteria, such as *Pantoea* and *Wolbachia*, likely reflecting its broader host range and greater exploitation of plant resources (Hirose et al., 2006; Barr et al., 2010; Graham and Wilson, 2012; Medina et al., 2018). In contrast, *P. guildinii* shows a higher prevalence of diapause-associated bacteria, particularly *Candidatus Benitsuchiphilus*, consistent with its narrower host range and a longer diapause period approximately five months longer than *E. heros* (Panizzi and Vivan, 1997; Zerbino et al., 2020). This association mirrors findings in *P. japonensis*, which undergoes diapause for up to two years and relies on *Candidatus Benitsuchiphilus* for uric acid metabolism (Mondal et al., 2020).

## 5 Concluding remarks

This study provides the first comprehensive characterization of the bacterial gut microbiota of two major soybean pests: the neotropical brown stink bug *Euschistus heros* and the redbanded stink bug *Piezodorus guildinii*. Our goal was to better understand insect–microbe associations that may contribute to the persistence and adaptability of these pests in soybean fields across the Americas.

We found no fixed pattern in gut microbiome composition across populations, as most microorganisms are environmentally acquired from host plants and soil. However, their persistence within the gut appears to be modulated by host genotype, which shapes the physiological environment and exerts selective pressures on microbial colonization. Both bacterial and fungal genera with known phytopathogenic potential were detected, some of which can likely be disseminated to crops through feeding, amplifying their agricultural impact.

In addition, bacterial genera associated with nutrient provisioning, phytochemical detoxification, and insecticide degradation are predicted to be associated with traits that could enhance the adaptive capacity of stink bugs, facilitating colonization of new hosts and survival under diverse management regimes. These findings provide a predictive framework for understanding why these pests are difficult to control and emphasize that a single, uniform management strategy is unlikely to be effective across all regions.

Future research should focus on elucidating the mechanisms of microbial acquisition and transmission, with particular attention to symbionts that mediate insect adaptation. Such symbionts could potentially be targeted or engineered as biocontrol agents, improving the sustainability and effectiveness of pest management strategies in soybean agroecosystems.

## Data Availability

The datasets presented in this study can be found in online repositories. The names of the repository/repositories and accession number(s) can be found at: https://www.ncbi.nlm.nih.gov/, PRJNA764175 https://www.ncbi.nlm.nih.gov/, PRJNA764176.

## Footnotes

1
https://benjjneb.github.io/dada2/tutorial.html
