# Supplementary material for: Host genetics and environment shape the gut microbiome of Euschistus heros and Piezodorus guildinii and potentially influencing their adaptation
Source: Front Microbiol. 2026 May 5;17:1782301. doi: 10.3389/fmicb.2026.1782301 (PMC13183665; doi:10.3389/fmicb.2026.1782301)
Supplement: Supplementary file 1 [file Supplementary_file_1.docx]

**Figure S1. (A)** Taxonomic composition of the bacterial gut microbiome at the genus level of *E. heros* collected in Brazil. **(B)** Taxonomic composition of the bacterial gut microbiome at the genus level of *P. guildinii* collected in Brazil and the United States. **(C)** Taxonomic composition of the bacterial gut microbiome at the genus level of *E. heros* and *P. guildinii* collected in Brazil.
